# Analysis of the association between history of gestational diabetes mellitus and hypertensive disorders in a subsequent pregnancy: a retrospective cohort study

**DOI:** 10.3389/fendo.2026.1736779

**Published:** 2026-03-12

**Authors:** Yuzhen Liu, Shilin Zhong, Kai Zhong, Lihua Su, Min Wang, Ying Wang, Juan Yang, Chang Xu, Yuqing Deng, Kaidong Ma, Yanmei Li

**Affiliations:** 1 Center of Obstetrics and Gynecology, Peking University Shenzhen Hospital, Shenzhen, Guangdong, China; 2 Shenzhen Peking University-Hong Kong University of Science and Technology (PKU-HKUST) Medical Center, Institute of Obstetrics and Gynecology, Shenzhen, Guangdong, China; 3 Peking University Shenzhen Hospital, Shenzhen Key Laboratory on Technology for Early Diagnosis of Major Gynecologic Diseases, Shenzhen, Guangdong, China; 4 Intelligent Hospital Research Academy, Peking University Shenzhen Hospital, Shenzhen, Guangdong, China; 5 Department of Obstetrics and Gynecology, Guangzhou Women and Children’s Medical Center, Guangzhou Medical University, Guangzhou, Guangdong, China

**Keywords:** gestational diabetes mellitus, hypertensive disorders of pregnancy, in the second pregnancy, pre-eclampsia, recurrence, stratified analysis, subsequent pregnancy

## Abstract

**Aim:**

To investigate the association between history of gestational diabetes mellitus (GDM) and the risk of hypertensive disorders of pregnancy in subsequent pregnancy (s-HDP).

**Methods:**

A retrospective cohort of 5, 928 women with two consecutive singleton deliveries was categorized by GDM status across pregnancies: GDM^-^/^-^ (none), GDM^+^/^-^ (GDM history without recurrence), GDM^-^/^+^ (only GDM in subsequent pregnancy), and GDM^+^/^+^ (GDM history with recurrence). The primary endpoint was the occurrence of s-HDP in subsequent pregnancy, with pre-eclampsia (s-PE) as a secondary endpoint. Multivariable regression assessed associations between GDM patterns and these endpoints. Stratified analysis was performed to identify high-risk subgroups.

**Results:**

Only GDM^+^/^+^ was significantly associated with increased s-HDP risk after full adjustment (*P*<0.05). This association remained significant in subgroups with prior HDP, long interpregnancy interval (LIPI, ≥36 months), advanced maternal age, or overweight/obesity. For s-PE, the association with GDM^+^/^+^ was attenuated after full adjustment (*P*>0.05) but remained significant in women with LIPI (*P*<0.05). Other GDM patterns showed no significant associations.

**Conclusion:**

A history of GDM is independently associated with increased HDP in a subsequent pregnancy only when GDM recurs, particularly among high-risk women. In contrast, a history of GDM without recurrence showed no significant association with s-HDP. The PE association was strongest in women with long interpregnancy intervals.

## 1 Introduction

Hypertensive disorders of pregnancy (HDP) and gestational diabetes mellitus (GDM) are two major metabolic complications that significantly contribute to maternal and perinatal morbidity and mortality worldwide (1). In China, the prevalence of HDP is approximately 7.3% (2) and has been steadily increasing (3). Concurrently, the prevalence of GDM has reached 14.8% (4), with a trend towards affecting younger women and an increasing incidence in China (5). Both conditions are not only associated with adverse pregnancy outcomes but also pose profound long-term risks for maternal cardiometabolic health and offspring well-being (6, 7).

Existing evidence confirms that GDM and HDP share core pathophysiological mechanisms. Insulin resistance (8), a key driver of GDM, synergizes with oxidative stress (9) and chronic inflammation (10) to induce endothelial dysfunction (11), which constitutes the pathological basis for the clinical manifestations in the second stage of HDP (12). Known risk factors for HDP also include history of pre-eclampsia (13), multiple pregnancy (14), pre-pregnancy diabetes (15), pre-pregnancy BMI (16) or obesity (17), primipara (18), and advanced maternal age (≥35 years) (19). Additionally, women with a history of GDM face a substantially increased long-term risk of hypertension (20). However, it remains unclear whether the risk of developing HDP in a subsequent pregnancy among women with prior GDM is influenced by their specific GDM history status, and stratified analyses based on patterns across two consecutive pregnancies are lacking. Crucially, the effect of previous GDM on HDP risk in a subsequent pregnancy should be examined in relation to whether GDM recurs in subsequent pregnancy.

In the context of China’s adjusted fertility policy, the proportion of multiparous and advanced-age pregnant women has risen significantly (21), further elevating the incidence of GDM and HDP and posing new challenges for antenatal care. Elucidating the association between different patterns of GDM across two pregnancies and the risk of HDP in a subsequent pregnancy is crucial for developing targeted risk prevention strategies and improving both short- and long-term maternal and neonatal outcomes. This study aims to address a critical knowledge gap by systematically investigating the association between different GDM history patterns across two consecutive pregnancies and the risk of HDP and its pre-eclampsia subtype in the subsequent pregnancy.

## 2 Materials and methods

### 2.1 Participants and study design

This retrospective cohort study enrolled women with two consecutive singleton deliveries at Peking University Shenzhen Hospital between Jan 2002 and Mar 2024. Participants were categorized into two groups based on hypertensive status in subsequent pregnancy: the s-HDP group comprised those diagnosed with HDP in subsequent pregnancy, while the s-NBP (normal blood pressure) group included those without HDP. Inclusion criteria were: (1) age between 16 and 50 years; (2) Chinese nationality; (3) in-hospital delivery; and (4) two consecutive pregnancies. Exclusion criteria included: (1) incomplete medical records; (2) pregnancy number other than two; (3) multiple gestation in either pregnancy. The study was approved by the Ethics Committee of Peking University Shenzhen Hospital (Approval Number: 2023-103-R1).

### 2.2 Diagnostic criteria and variable definitions

The diagnosis of HDP followed the “Diagnosis and treatment of hypertension and pre-eclampsia in pregnancy: a clinical practice guideline in China (2020)” (22). Preeclampsia was diagnosed in pregnant women after 20 weeks of gestation who presented with new-onset hypertension (systolic BP ≥140 mmHg and/or diastolic BP ≥90 mmHg) and met at least one of the following criteria: 1) proteinuria, defined as a 24-hour urinary protein excretion >0.3 g, a urine protein/creatinine ratio >0.3, or a random urine protein ≥1+ (in the absence of quantitative testing); or 2) in the absence of proteinuria, evidence of new-onset systemic involvement affecting organs such as the cardiac, pulmonary, hepatic, renal, hematological, digestive, or neurological systems, or placental-fetal complications (22).

GDM was diagnosed according to the evolving clinical guidelines during the study period. For deliveries prior to 2010, a two-step approach was implemented: all pregnant women underwent a 50g glucose challenge test (GCT) at 24–28 weeks of gestation, and those with a 1-hour glucose level ≥7.8 mmol/L proceeded to a 100g oral glucose tolerance test (100g-OGTT). GDM was diagnosed if two or more of the following thresholds were met or exceeded: fasting ≥5.8 mmol/L, 1-hour ≥10.6 mmol/L, 2-hour ≥9.2 mmol/L, or 3-hour ≥8.1 mmol/L. From 2010 onward, the International Association of the Diabetes and Pregnancy Study Groups (IADPSG) criteria (23) were adopted, employing a one-step 75g oral glucose tolerance test (75g-OGTT) at 24–28 weeks. Under these criteria, GDM was diagnosed if any one of the following thresholds was met: fasting plasma glucose ≥5.1 mmol/L, 1-hour plasma glucose ≥10.0 mmol/L, or 2-hour plasma glucose ≥8.5 mmol/L.

Based on the GDM status in the two consecutive pregnancies, participants were categorized into four mutually exclusive groups: GDM^-^/^-^ (no GDM in either pregnancy), GDM^+^/^-^ (GDM history without recurrence), GDM^-^/^+^ (GDM only in subsequent pregnancy), and GDM^+^/^+^ (GDM history with recurrence).

Other variables were defined and classified as follows: (1) Body mass index (BMI) was categorized according to WHO standards: underweight (UW, <18.5 kg/m²), normal weight (NW, 18.5–23.9 kg/m²), and overweight or obesity (OB, ≥24 kg/m²) (24). (2) Preterm birth (PTB) was defined as delivery occurring before 37 gestational weeks. (3) Interpregnancy interval (IPI) was calculated as the time interval between the delivery date of the first pregnancy and the conception date of the subsequent pregnancy (25), and was categorized into short IPI (SIPI, <36 months) and long IPI (LIPI, ≥36 months). (4) Advanced maternal age (AMA) was defined as maternal age in subsequent pregnancy ≥35 years. The prefixes “f-” and “s-” denote variables related to the first and subsequent pregnancy, respectively.

### 2.3 Data collection

Data were extracted from the hospital medical record and the Shenzhen Maternal and Child Health Care System, including maternal age, parity, BMI, IPI, GDM, HDP, PE, PTB, and cesarean section (CS) for both pregnancies.

### 2.4 Statistical analysis

Statistical analyses were performed using SPSS 25.0 software (IBM Corp., Armonk, NY, USA). (1) Descriptive analysis: Categorical variables were presented as frequency and percentage [n (%)]. Continuous variables with normal distribution were described as mean ± standard deviation (x ± s), while non-normally distributed variables were reported as median and interquartile range [median (Q1-Q3)]. (2) Univariate analysis: Group comparisons for categorical variables were conducted using the Chi-square test. Normally distributed continuous variables were compared using the independent samples t-test, and non-normally distributed variables were compared using the Mann-Whitney *U* test. (3) Multivariate analysis: Binary logistic regression models were employed with s-HDP (or s-PE) as the dependent variable. Two models were constructed: Model 1 adjusted for first-pregnancy related factors (GDM patterns, LIPI, f-HDP, f-PTB, f-CS), which are known to be associated with subsequent pregnancy hypertension. Model 2 further included key subsequent-pregnancy characteristics (s-AMA, s-BMI categories [s-UW, s-NW, s-OB; reference: s-NW], s-parity) in addition to all Model 1 variables. First-pregnancy BMI (f-BMI) was not included in the multivariate models due to a high proportion of missing data (42.0%) and its strong correlation with the more proximate and complete measure, s-BMI, which was retained as a more relevant risk factor for the index pregnancy. Variance inflation factor (VIF) analysis confirmed the absence of severe multicollinearity in the final models (all VIFs < 3, **Supplementary Table 1**). (4) Stratified analysis: Stratified analyses were performed by f-HDP (yes/no), s-AMA (yes/no), IPI category (SIPI/LIPI), and s-BMI category (s-UW/s-NW/s-OB) to examine the association between GDM categories and s-HDP (and s-PE) within each subgroup. All statistical tests were two-sided, and a P-value <0.05 was considered statistically significant.

## 3 Results

### 3.1 General characteristics of the study population

**Figure 1:**
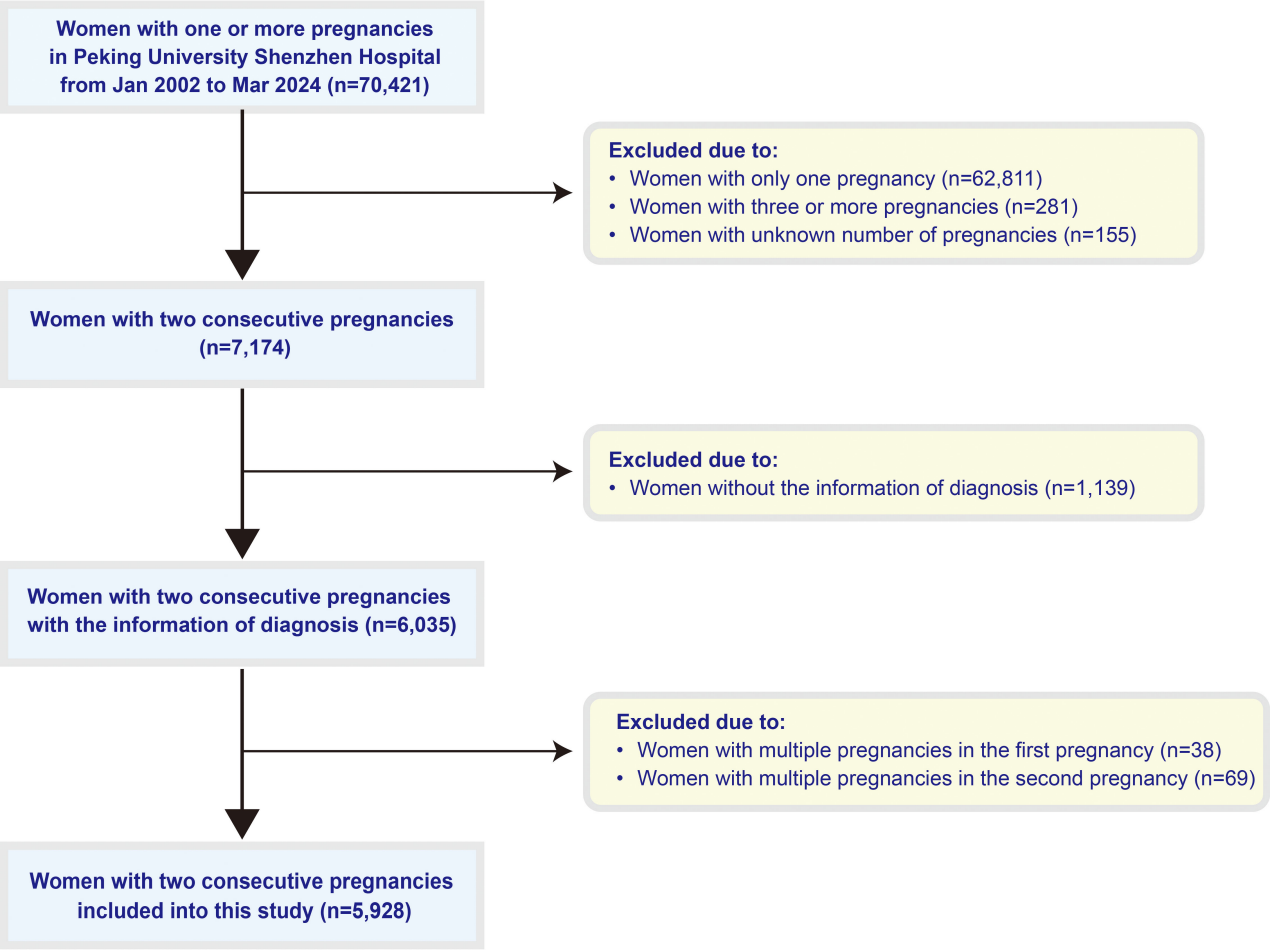
Flowchart of study inclusion and exclusion.

A total of 5, 928 women with two consecutive singleton pregnancies and deliveries were enrolled in this study. The flow of participant screening is detailed in **Figure 1**.

In the first pregnancy, 558 women (9.41%) were diagnosed with GDM, and 182 (3.07%) had f-HDP. Among the f-HDP cases, 64 (1.08%) were gestational hypertension (f-GH) and 118 (1.99%) were pre-eclampsia (f-PE). Additionally, 316 women (5.33%) experienced preterm birth (f-PTB), 2, 277 (38.41%) underwent cesarean section (f-CS), and 2,898(48.89%) had a long interpregnancy interval (LIPI). In subsequent pregnancy, the prevalence of GDM was 15.62% (n=926), and the prevalence of s-HDP was 2.19% (n=130). Among these 130 s-HDP cases, 69 were diagnosed with pre-eclampsia (s-PE), constituting 1.16% of the total study population and 53.08% of all s-HDP cases. Furthermore, 1, 612 women (27.19%) were of advanced maternal age (s-AMA), and 916 (15.45%) were classified as overweight or obese (s-OB).

Regarding the distribution of GDM status across both pregnancies, 4, 753 women (80.18%) had no GDM in either pregnancy (GDM^-^/^-^), 249 (4.20%) had GDM history without recurrence (GDM^+^/^-^), 617 (10.41%) had GDM only in subsequent pregnancy (GDM^-^/^+^), and 309 (5.21%) had GDM history with recurrence (GDM^+^/^+^).

A comparison of baseline characteristics between the s-HDP and s-NBP groups (**Table 1**) revealed that women in the s-HDP group had significantly higher f-MA, s-MA, s-BMI, and IPI, alongside significantly lower gestational week at delivery (GWD) and neonatal birth weight (NBW) in both pregnancies (all P<0.05). The proportions of women with GDM^+^/^+^, f-HDP (and its subtypes f-GH and f-PE), f-PTB, f-CS, LIPI, s-AMA, s-OB, s-PTB and s-CS were all significantly higher in the s-HDP group (all P<0.05), while the proportions of GDM^-^/^-^ and s-NW were significantly lower.

**Table 1:** Comparison of risk factors between the s-HDP and s-NBP groups.

Risk factors	s-HDP (n=130)	s-NBP (n=5798)	*t/Z/*Chi-square	*P*
Continuous variables				
f-MA (years)	29.0 (27.0-31.0)	28.0 (26.0-30.0)	3.074	0.002
s-MA (years)	34.0 (31.0-35.0)	32.0 (29.0-34.0)	4.873	<0.001
s-BMI (kg/m^2^)	23.83 (21.50-26.66)	20.90 (19.26-22.91)	7.265	<0.001
s-parity	2 (2-2)	2 (2-2)	1.619	0.106
IPI (months)	46.56 (27.08-70.46)	35.26 (20.63-57.30)	3.926	<0.001
f-GWD	39.0 (37.00-39.00)	39.0 (38.00-40.00)	4.435	<0.001
f-NBW	3150.00 (2800.0-3412.5)	3250.0 (3000.0-3550.0)	3.349	0.001
s-GWD	38.00 (36.0-39.0)	39.00 (38.0-39.0)	8.574	<0.001
s-NBW	3000.0 (2350.0-3450.0)	3300.0 (3050.0-3550.0)	6.357	<0.001
Categorical variables				
GDM patterns [n(%)]				
GDM^-^/^-^	85 (65.4)	4668 (80.5)	18.305	<0.001
GDM^+^/^-^	5 (3.8)	244 (4.2)	0.041	0.839
GDM^-^/^+^	19 (14.6)	598 (10.3)	2.523	0.112
GDM^+^/^+^	21 (16.2)	28 (5.0)	32.204	<0.001
f-HDP [n(%)]				
Yes	55 (42.3)	127 (2.2)	687.631	<0.001
No	75 (57.7)	5671 (97.8)		
f-GH [n(%)]				
Yes	18 (13.8)	46 (0.8)	202.844	<0.001
No	112 (86.2)	5752 (99.2)		
f-PE [n(%)]				
Yes	37 (28.5)	81 (1.4)	477.388	<0.001
No	93 (71.5)	5717 (98.6)		
f-PTB [n(%)]				
Yes	21 (16.2)	295 (5.1)	30.853	<0.001
No	109 (83.8)	5503 (94.9)		
f-CS [n(%)]				
Yes	75 (57.7)	2202 (38.0)	20.888	<0.001
No	55 (42.3)	3596 (62.0)		
IPI categories [n(%)]				
SIPI	46 (35.4)	2984 (51.5)	13.146	<0.001
LIPI	84 (64.6)	2814 (48.5)		
s-MA categories [n(%)]				
s-YMA	72 (55.4)	4244 (73.2)	20.378	<0.001
s-AMA	58 (44.6)	1554 (26.8)		
s-BMI [n(%)]				
s-UW	9 (7.3)	720 (13.2)	3.629	0.057
s-NW	60 (48.8)	3887 (71.1)	28.792	<0.001
s-OB	54 (43.9)	862 (15.8)	69.548	<0.001
s-PTB [n (%)]				
Yes	37 (28.5)	305 (5.3)	125.898	<0.001
No	93 (71.5)	5493 (94.7)		
s-CS[n (%)]				
Yes	93 (71.5)	2498 (43.1)	41.842	<0.001
No	37 (28.5)	3300 (56.9)		
s-MAC[n (%)]				
Yes	9 (6.9)	322 (5.6)	0.452	0.501
No	121 (93.1)	5476 (94.4)		

f-, in the first pregnancy; s-, in subsequent pregnancy; MA, maternal age; BMI, body mass index; IPI, interpregnancy interval; GWD, gestational week of delivery; NBW, neonatal birth weight; PTB, preterm birth; CS, cesarean section; MAC, macrosomia; GDM, gestational diabetes mellitus; GDM^-^/^-^, no GDM in either pregnancy; GDM^+^/^-^, GDM history without recurrence; GDM^-^/^+^, GDM only in subsequent pregnancy; GDM^+^/^+^, GDM history with recurrence; NBP, normal blood pressure; HDP, hypertensive disorders of pregnancy; GH, gestational hypertension; PE, pre-eclampsia; PTB, preterm birth; CS, cesarean section; SIPI, short interpregnancy interval (<36 months); LIPI, long interpregnancy interval (≥36 months); UW, underweight; NW, normal weight; OB, overweight/obesity.

### 3.2 Association between GDM patterns and s-HDP

The results of the unadjusted and adjusted logistic regression analyses for the association between GDM patterns and s-HDP are presented in **Table 2**. In the unadjusted model, both GDM^-^/^+^ (OR = 1.745, 95% CI: 1.053–2.890) and GDM^+^/^+^ (OR = 4.004, 95% CI: 2.448–6.551) were significantly associated with s-HDP compared to the GDM^-^/^-^ reference group. After adjustment for first-pregnancy factors in Model 1, only GDM^+^/^+^ remained significantly associated with s-HDP (OR = 2.729, 95% CI: 1.555-4.791). In the fully adjusted Model 2 (which included both first and subsequent-pregnancy factors), the association for GDM^+^/^+^ persisted, though slightly attenuated (OR = 2.272, 95% CI: 1.271-4.062). Neither GDM^+^/^-^ nor GDM^-^/^+^ showed a significant association with s-HDP in any of the adjusted models. A sensitivity analysis was conducted in 3, 447 women with complete s-GWG data to assess its influence. After additional adjustment for s-GWG, the association between GDM^+^/^+^ and s-HDP remained significant (aOR=2.844, 95% CI: 1.471-5.497). In contrast, s-GWG itself was not significantly associated with s-HDP in the overall cohort (**Supplementary Table 2**).

**Table 2:** Impact of GDM status across two pregnancies on s-HDP in unadjusted and adjusted models.

GDM patterns	unadjusted OR (95% CI)	adjusted OR (95% CI) in Model 1	adjusted OR (95% CI) in Model 2
GDM^-^/^-^	reference	Reference	Reference
GDM^+^/^-^	1.125 (0.453-2.799)	1.089 (0.414-2.868)	0.966 (0.359-2.601)
GDM^-^/^+^	**1.745 (1.053-2.890)**	1.149 (0.661-1.997)	0.987 (0.556-1.751)
GDM^+^/^+^	**4.004 (2.448-6.551)**	**2.729 (1.555-4.791)**	**2.272 (1.271-4.062)**

GDM, gestational diabetes mellitus; GDM^-^/^-^, no GDM in either pregnancy; GDM^+^/^-^, GDM history without recurrence; GDM^-^/^+^, GDM only in subsequent pregnancy; GDM^+^/^+^, GDM history with recurrence; HDP, hypertensive disorders of pregnancy; OR, odds ratio; CI, confidence interval. Model 1: Adjusted for first-pregnancy factors (GDM patterns, LIPI, f-HDP, f-PTB, f-CS). Model 2: Adjusted for factors in Model 1 plus subsequent-pregnancy factors (s-AMA, s-BMI categories, s-parity). Numbers with statistical significance were marked in bold.

### 3.3 Stratified analysis of the association between GDM patterns and s-HDP

**Figure 2:**
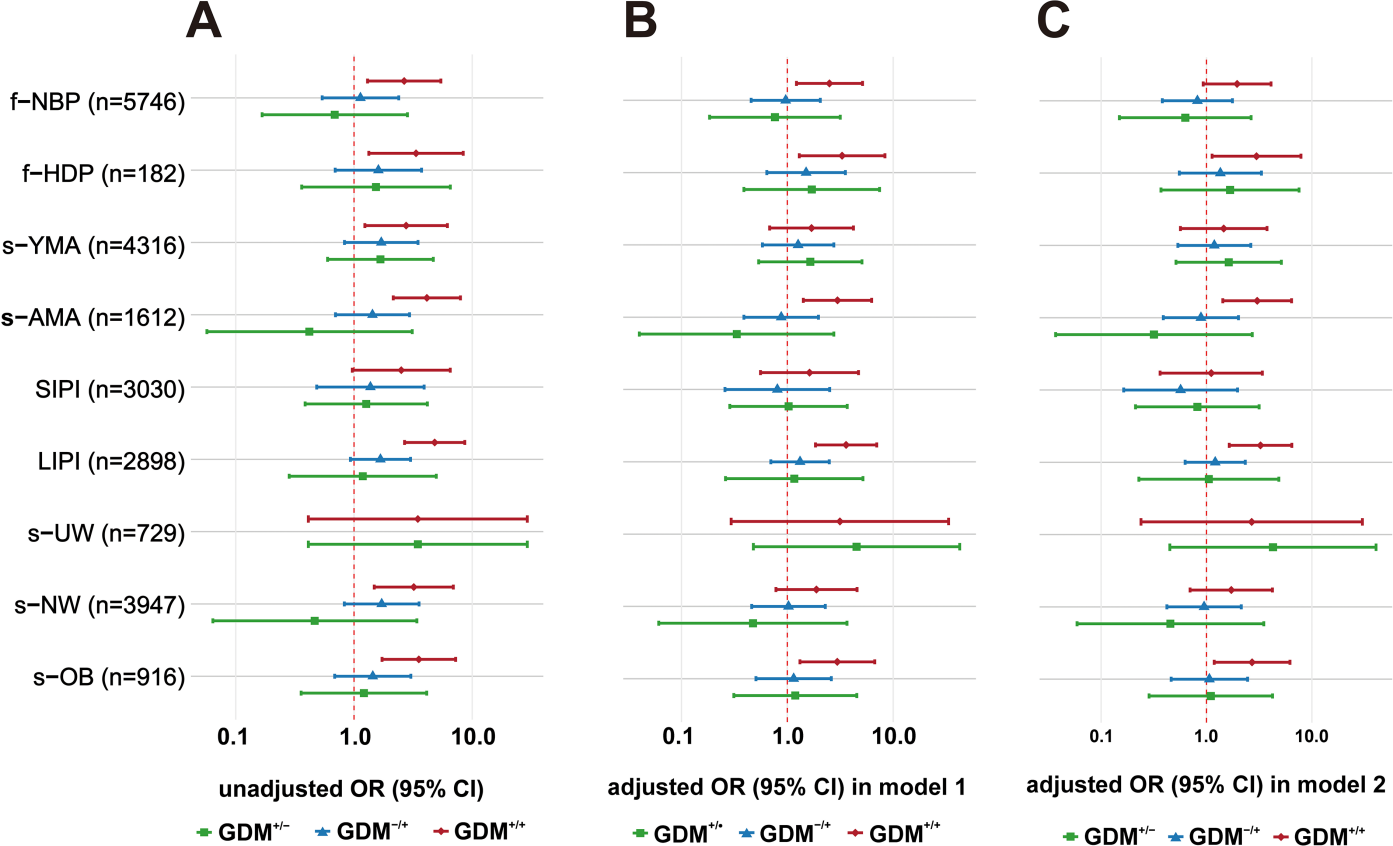
Stratified analysis of the association between GDM patterns and HDP in subsequent pregnancy across sequential adjustment models. The reference group was GDM^-^/^-^. Analyses are presented for: **(A)** the unadjusted model, **(B)** Model 1 (adjusted for first-pregnancy factors: GDM patterns, LIPI, f-HDP, f-PTB, f-CS), and **(C)** Model 2 (adjusted for factors in Model 1 plus subsequent-pregnancy factors: s-AMA, s-BMI categories, s-parity). f-, first pregnancy; s-, subsequent pregnancy; NBP, normal blood pressure; HDP, hypertensive disorders of pregnancy; YMA, young maternal age; AMA, advanced maternal age; SIPI, short interpregnancy interval; LIPI, long interpregnancy interval; UW, underweight; NW, normal weight; OB, overweight/obesity.

Stratified analyses across key patient subgroups revealed a robust and evolving association between GDM history with recurrence (GDM^+^/^+^) and s-HDP, as detailed in **Figure 2**. In the unadjusted analysis (**Figure 2A**), GDM^+^/^+^ was significantly associated with an elevated risk of s-HDP across most high-risk subgroups, including women with a prior history of HDP (f-HDP), those with a long interpregnancy interval (LIPI), advanced maternal age (s-AMA), or overweight/obesity (s-OB) in subsequent pregnancy. This association persisted, though with some attenuation, after adjustment for first-pregnancy factors in Model 1 (**Figure 2B**), remaining significant in the f-HDP, LIPI, s-AMA, and s-OB subgroups. Crucially, in the fully adjusted Model 2 (**Figure 2C**), which accounted for both first and subsequent-pregnancy characteristics, the significant association between GDM^+^/^+^ and s-HDP endured in these same high-risk subgroups, underscoring the independent effect of recurrent GDM. In contrast, the association was not significant in subgroups with a short interpregnancy interval (SIPI) or underweight (s-UW) in any model. Across all analyses, neither GDM occurring only in the first (GDM^+^/^-^) nor only in subsequent pregnancy (GDM^-^/^+^) demonstrated significant associations with s-HDP. Stratified analyses restricted to the 3, 447 women with complete s-GWG data demonstrated that the significant association between GDM^+^/^+^ and s-HDP persisted in the LIPI, s-AMA, and s-OB subgroups. In contrast, s-GWG itself was not significantly associated with s-HDP in most subgroups, with the sole exception of women with a history of HDP in their first pregnancy (f-HDP), in whom higher s-GWG was unexpectedly associated with a lower risk of s-HDP (**Supplementary Table 2**).

### 3.4 Association between GDM patterns and pre-eclampsia in subsequent pregnancy

We performed a parallel analysis focusing specifically on pre-eclampsia in subsequent pregnancy (s-PE). The baseline characteristics of the s-PE group (n=69) compared to the non-pre-eclampsia group (s-nonPE, n=5, 859) are shown in **Supplementary Table 3**. Women who developed s-PE had significantly higher median f-MA, s-MA, s-BMI, and IPI (all P<0.01). The proportions of GDM^+^/^+^, f-HDP (and its subtypes f-GH and f-PE), f-PTB, f-CS, LIPI, s-AMA, and s-OB were all significantly higher in the s-PE group (all P<0.05).

The results of the regression analyses for s-PE are summarized in **Table 3**. In the unadjusted model, GDM history with recurrence (GDM^+^/^+^) was significantly associated with an increased risk of s-PE (OR = 3.278, 95% CI: 1.642–6.544). After adjustment for first-pregnancy factors in Model 1, the association was attenuated and was no longer statistically significant (OR = 2.008, 95% CI: 0.954–4.226). This attenuation continued in the fully adjusted Model 2, which further accounted for subsequent-pregnancy factors (OR = 1.686, 95% CI: 0.778–3.654). No other GDM patterns (GDM^+^/^-^ or GDM^-^/^+^) showed significant associations with s-PE in any of the models. In the sensitivity analysis which was conducted in 3, 447 women with complete s-GWG data to assess its influence. After additional adjustment for s-GWG, the association between GDM^+^/^+^ and s-PE remained not significant (aOR=1.231, 95% CI: 0.471-3.220) (**Supplementary Table 4**).

**Table 3:** Association between GDM patterns across two pregnancies and s-PE.

GDM patterns	Unadjusted OR (95% CI)	Adjusted OR (95% CI) in model 1	Adjusted OR (95% CI) in model 2
GDM^-^/^-^	Reference	reference	Reference
GDM^+^/^-^	1.195 (0.370-3.865)	1.202 (0.359-4.025)	1.051 (0.303-3.651)
GDM^-^/^+^	1.288 (0.606-2.735)	0.801 (0.364-1.763)	0.666 (0.292-1.519)
GDM^+^/^+^	**3.278 (1.642-6.544)**	2.008 (0.954-4.226)	1.686 (0.778-3.654)

GDM, gestational diabetes mellitus; GDM^-^/^-^, no GDM in either pregnancy; GDM^+^/^-^, GDM history without recurrence; GDM^-^/^+^, GDM only in subsequent pregnancy; GDM^+^/^+^, GDM history with recurrence; PE, pre-eclampsia; OR, odds ratio; CI, confidence interval. Model 1: Adjusted for first-pregnancy factors (GDM patterns, LIPI, f-HDP, f-PTB, f-CS). Model 2: Adjusted for factors in Model 1 plus subsequent-pregnancy factors (s-MA, s-BMI, s-parity). Numbers with statistical significance were marked in bold.

### 3.5 Stratified analysis of the association between GDM patterns and s-PE

Stratified analysis for s-PE provided further insights (**Supplementary Table 5**). In the unadjusted model, significant associations between GDM^+^/^+^ and s-PE were observed in subgroups with s-AMA, LIPI, and s-OB. After adjustment for first-pregnancy factors (Model 1), the association remained significant only in the LIPI subgroup. Notably, in the fully adjusted model (Model 2), which accounts for both first and subsequent-pregnancy characteristics, the significant association between recurrent GDM and s-PE persisted only in the subgroup of women with LIPI (OR = 2.486, 95% CI: 1.076-5.746). In stratified analyses restricted to the 3, 447 women with complete s-GWG data, the association between GDM^+^/^+^ and s-PE remained non-significant in most subgroups after additional adjustment for s-GWG. A significant association was observed only in the LIPI subgroup after adjustment for first-pregnancy factors (Model 1: OR = 3.096, 95% CI: 1.234–7.767); however, this association was attenuated and lost statistical significance in the fully adjusted Model 2 (OR = 1.879, 95% CI: 0.699–5.051) (**Supplementary Table 4**). Notably, s-GWG itself was not significantly associated with s-PE in any model or subgroup (**Supplementary Table 4**).

## 4 Discussion

This large retrospective cohort study, comprising 5, 928 women with two consecutive singleton pregnancies, provides compelling evidence that GDM history with recurrence (GDM^+^/^+^) is an independent risk factor for hypertensive disorders of pregnancy (HDP) in a subsequent pregnancy. The key finding is that this association is specific to the recurrent GDM pattern and is not observed when GDM history does not recur in subsequent pregnancy (GDM^+^/^-^). Furthermore, our study delineates that this risk is not uniform but is concentrated in specific high-risk subgroups and demonstrates a distinct pattern for pre-eclampsia compared to overall HDP.

The robust and stratified association between recurrent GDM and s-HDP merits detailed consideration. Our findings that the GDM^+^/^+^-s-HDP association was particularly strong and persistent in women with a history of HDP (f-HDP), those with a long interpregnancy interval (LIPI), advanced maternal age (s-AMA), or overweight/obesity (s-OB) illuminate a pattern of risk accumulation and synergy. In the f-HDP subgroup, the significant association suggests a powerful additive effect. Women with prior HDP are known to have pre-existing vascular endothelial dysfunction and are at the highest risk for recurrence (26). The presence of recurrent GDM, indicative of persistent metabolic dysfunction (27), likely exacerbates this underlying vascular vulnerability (28), creating a compounded pathological burden that significantly elevates s-HDP risk. Similarly, the significant association observed in the s-AMA subgroup aligns with the physiological decline in vascular elasticity (29) and insulin sensitivity (30) associated with aging. Recurrent GDM in these women appears to compound these age-related risks, further increasing susceptibility to HDP (31). For the LIPI subgroup, a prolonged interval (≥36 months) may facilitate the accumulation of metabolic burden, such as weight gain (32), or a decline in overall cardiometabolic health (33), which then interacts synergistically with the persistent dysmetabolic state of recurrent GDM. The stark contrast with the non-significant finding in the SIPI subgroup underscores that a shorter interval may not provide sufficient time for such risk factors to accumulate to a pathogenic threshold. Finally, the significant association in the s-OB subgroup, and its absence in normal and underweight women, strongly reinforces the central role of adiposity. Overweight/obesity is a well-established driver of insulin resistance (34) and chronic inflammation (35), and its co-existence with recurrent GDM creates a potent “second-hit” scenario, further aggravating vascular damage and firmly establishing the observed association (36).

The observed association between recurrent GDM and s-HDP can be explained by shared pathophysiological pathways between GDM and HDP, which include insulin resistance (11), chronic low-level inflammation (10), and angiogenic imbalance (37) as the core pathogenic links. GDM^+^/^+^ indicates that the pregnant woman has persistent abnormal glucose metabolism. This persistence may stem from the ineffective correction of insulin resistance during the interval between two pregnancies (38). Insulin resistance not only activates the renin-angiotensin-aldosterone system but also increases the sensitivity of vascular smooth muscle to vasoconstrictive substances, thereby promoting an increase in blood pressure (39). Meanwhile persistent GDM state is often accompanied by chronic inflammatory responses. Inflammatory factors such as tumor necrosis factor-α (TNF-α) and interleukin-6 (IL-6) can damage vascular endothelial cells, reduce nitric oxide production, and lead to decreased vascular dilation function, laying the pathological foundation for the occurrence of HDP (40). Furthermore, the recurrence of GDM is closely related to overweight/obesity (38). It can further aggravate insulin resistance and vascular damage through the imbalance of leptin and adiponectin secreted by adipose tissue (41), forming a vicious cycle of “GDM recurrence - obesity - inflammation - HDP” (42). This also explains why the association between GDM^+^/^+^ and s-HDP is more significant in the s-OB subgroup. In contrast, GDM^+^/^-^ (single pregnancy with GDM) has no significant association with s-HDP. This might be because these pregnant women, during the interval between two pregnancies, underwent lifestyle interventions such as dietary control and exercise (43), or received necessary drug treatment (44), which corrected the abnormal glucose metabolism and insulin resistance, thus cutting off the “GDM-HDP” pathological chain. While GDM^-^/^+^ (subsequent pregnancy with GDM) has no significant association. This might be because the diagnosis of the subsequent GDM occurred between 24 and 28 weeks of pregnancy, and the diagnosis was relatively late, so it had not yet had a continuous impact on blood pressure, or a single exposure to GDM was not sufficient to trigger long-term pathological changes in the vascular system.

When focusing specifically on pre-eclampsia (s-PE), a more severe and etiologically distinct subtype of HDP, the association with recurrent GDM presented a more nuanced picture. In the unadjusted model, GDM^+^/^+^ was significantly associated with an elevated risk of s-PE. However, the association lost statistical significance after controlling for first-pregnancy factors (Model 1) and was further attenuated in the fully adjusted model that additionally accounted for subsequent-pregnancy characteristics such as maternal age and BMI (Model 2). This attenuation suggests that the link between recurrent GDM and PE is substantially confounded by, or mediated through, the metabolic and physiological profile of the woman at the time of her subsequent pregnancy. Notwithstanding this overall finding, the stratified analysis yielded a critical insight: the significant association between recurrent GDM and s-PE persisted independently in the subgroup of women with a long interpregnancy interval (LIPI), even after full adjustment. This indicates that a prolonged interval between pregnancies is a potent effect modifier, potentially creating a window for the accumulation of metabolic risk factors or a decline in vascular health (45). This state may then synergize with the persistent dysmetabolic burden signified by recurrent GDM, culminating in a significantly elevated risk of PE. The loss of significance in other high-risk subgroups like s-AMA and s-OB in the final model underscores that the risk conveyed by recurrent GDM for PE is not as broad as it is for overall HDP and is most potent under specific conditions, with a long interpregnancy interval being a predominant one (46). The smaller number of s-PE cases (n=69) compared to s-HDP (n=130) also implies that the analysis for PE had less statistical power, which likely contributes to the wider confidence intervals observed. Therefore, the non-significant finding in the overall fully adjusted model for PE should be interpreted as an indication of a more context-dependent relationship rather than evidence of no association.

The findings of this study align with several previous reports while further clarifying the differential effects of GDM history based on recurrence status. For instance, Wainstock et al. reported that first-pregnancy complications (including GDM) were associated with an elevated risk of subsequent pre-eclampsia (OR = 2.12, 95% CI: 1.72–2.61, *P* < 0.001) (47). However, that study excluded women with pre-eclampsia in their first pregnancy, limiting its generalizability to the population with prior HDP. Another earlier study also indicated that recurrent GDM is linked to an increased risk of HDP (38), though it neither used the GDM^-^/^-^ group as reference nor differentiated GDM patterns across consecutive pregnancies. More recently, a study published in AJOG suggested that a history of GDM, even in the absence of recurrence, may elevate the risk of pre-eclampsia (48). Although the AJOG study adopted the appropriate GDM^-^/^-^ reference, it did not adjust for prior HDP or PE in its multivariate analysis. The discrepancy between its results and ours—which specifically tie s-HDP risk to recurrent GDM—may stem from two factors: the lack of adjustment for key confounders such as prior HDP in the AJOG study, and the relatively limited number of s-PE cases (n=69) in our cohort, which may have reduced statistical power to detect a significant association in the fully adjusted model. The principal innovation of our study lies in its systematic classification of GDM status across two consecutive pregnancies into four distinct categories. This approach allowed us to demonstrate that the increased risk of s-HDP is specifically attributable to GDM history with recurrence (GDM^+^/^+^), rather than to a general history of GDM.

It is important to interpret these associations with caution regarding causality. While our models adjusted for a comprehensive set of available clinical and demographic confounders, the retrospective design precluded the collection of key metabolic and behavioral variables, such as detailed glycemic control measures (e.g., HbA1c, fasting glucose trends), insulin therapy during GDM pregnancies, gestational weight gain (GWG), and specific lifestyle factors (e.g., diet quality, physical activity). These unmeasured factors are known to influence both GDM recurrence and HDP risk and thus represent potential sources of residual confounding. Consequently, the observed associations, although robust within our adjusted models, should be viewed as identifying an important clinical risk pattern rather than providing definitive proof of a direct causal pathway. Future prospective studies incorporating serial metabolic assessments are needed to elucidate the precise mechanistic links and to determine the extent to which modifiable factors like GWG or glycemic management mediate the risk observed here.

To enhance methodological transparency, we note that our modeling strategy was guided by *a priori* clinical reasoning. Key continuous variables—including subsequent-pregnancy BMI, maternal age, and interpregnancy interval—were intentionally categorized using clinically meaningful thresholds (e.g., Asian-specific BMI cut-offs, advanced maternal age ≥35 years, long interpregnancy interval ≥36 months). This approach was chosen to optimize the clinical interpretability of effect estimates and to maintain consistency with the stratified analyses, where these same categorical definitions were used. Furthermore, we performed collinearity diagnostics for all multivariable models, and variance inflation factors were all below 3, confirming that multicollinearity did not compromise the stability of our final models.

It is necessary to objectively view the strengths and limitations of this study: In terms of strengths, the study adopted a cohort design of two consecutive singleton pregnancies, avoiding the problem of “recall bias of previous GDM status” in single-pregnancy studies; by subdividing GDM types and using GDM^-^/^-^ as the reference, the independent effect of GDM recurrence was clearly defined; through univariate, multivariate and stratified analyses, key confounding factors such as age, BMI, IPI, and f-HDP were comprehensively controlled, resulting in relatively high reliability of the results. The limitations include: First, as a retrospective study, no *a priori* sample size or power calculation was performed, and the final sample was determined by data availability within the specified period; while the overall cohort was substantial, this design may limit the power to detect smaller effect sizes, particularly in subgroup analyses. Second, some early cases were excluded due to the lack of pre-pregnancy BMI, gestational weight gain (GWG), and other data, which may introduce selection bias. Third, lifestyle factors (such as dietary structure, exercise frequency), metabolic indicators (such as fasting blood glucose, lipid levels), and medication use during the interval between the two pregnancies were not collected, making it impossible to further analyze the potential modifying effects of these factors on the association between GDM recurrence and s-HDP. Fourth, some subgroups (such as s-UW) and the number of s-PE cases were relatively small, which may lead to insufficient statistical power and require verification in larger samples. Fifth, we lacked direct glycemic measurements (such as OGTT results) during the interpregnancy interval to confirm normoglycemia in women with recurrent GDM (GDM^+^/^+^). Although routine clinical practice at our center includes postpartum OGTT follow-up and early pregnancy screening for pre-gestational diabetes, the absence of systematically recorded data means we cannot entirely exclude the possibility of undetected persistent metabolic dysfunction in this group. This represents a potential source of misclassification bias inherent to retrospective studies. Finally, all study subjects were from a single center within a specific urban Chinese population, which may introduce regional and demographic limitations. The use of standardized yet locally implemented diagnostic criteria and population-specific characteristics (such as a lower average BMI and the application of Asian-specific BMI cut-offs) may affect the generalizability of the absolute risk estimates and the observed strength of associations to other ethnic groups or healthcare settings with differing anthropometric norms and screening practices. Therefore, while the identified pattern of risk related to recurrent GDM is likely to be biologically informative, its quantitative implications and the relative importance of specific high-risk subgroups should be validated in multi-center, large-sample cohorts across diverse populations.

## 5 Conclusions

Against the backdrop of China’s adjusted fertility policy and the rising proportion of multiparous and advanced-age pregnancies, the incidences of GDM and HDP have been increasing. This study identifies GDM history with recurrence (GDM^+^/^+^) as a strong and independent clinical marker associated with an increased risk of HDP in subsequent pregnancies, particularly among women with additional risk factors such as prior HDP, advanced maternal age, long interpregnancy interval, or overweight/obesity. Given the potential for residual confounding, this pattern underscores a high-risk subgroup warranting intensified surveillance rather than confirming a direct causal effect. Clinical vigilance should include early and intensified blood pressure monitoring, advanced screening for GDM recurrence, and strict weight management. In conclusion, while GDM history without recurrence showed no significant association, the recurrent pattern provides an evidence-based foundation for risk stratification and targeted prevention strategies to improve maternal-infant outcomes.

## Data Availability

The raw data supporting the conclusions of this article will be made available by the authors, without undue reservation.
