# Supplementary material for: Analysis of the association between history of gestational diabetes mellitus and hypertensive disorders in a subsequent pregnancy: a retrospective cohort study
Source: Front Endocrinol (Lausanne). 2026 Mar 12;17:1736779. doi: 10.3389/fendo.2026.1736779 (PMC13017284; doi:10.3389/fendo.2026.1736779)
Supplement: Supplementary file 1 [file Table1.docx]

**Supplementary Table 1** Collinearity analysis of the independent variables

| variables | tolerance | VIF |
| --- | --- | --- |
| GDM patterns | 0.966 | 1.036 |
| LIPI | 0.876 | 1.142 |
| f-HDP | 0.958 | 1.043 |
| f-PTB | 0.975 | 1.026 |
| f-CS | 0.944 | 1.060 |
| s-AMA | 0.861 | 1.161 |
| s-BMI categories | 0.981 | 1.019 |
| s-parity | 0.986 | 1.014 |

f: in the first pregnancy; s: in the second pregnancy; GDM, gestational diabetes mellitus; LIPI, long interpregnancy interval; HDP, hypertensive disorders of pregnancy; PTB, preterm birth; CS, cesarean section; AMA, advanced maternal age; BMI, body mass index; VIF: variance inflation factor.
