# Supplementary material for: Analysis of the association between history of gestational diabetes mellitus and hypertensive disorders in a subsequent pregnancy: a retrospective cohort study
Source: Front Endocrinol (Lausanne). 2026 Mar 12;17:1736779. doi: 10.3389/fendo.2026.1736779 (PMC13017284; doi:10.3389/fendo.2026.1736779)
Supplement: Supplementary file 2 [file Table2.docx]

**Supplementary Table 2 Impact of s-GWG on s-HDP in unadjusted and adjusted models**

| The analyzed population | Independent variable | unadjusted OR (95% CI) | adjusted OR (95% CI) in Model 1 | adjusted OR (95% CI) in Model 2 |
| --- | --- | --- | --- | --- |
| All | GDM⁺/⁺ | - | **4.191(2.374-7.399)** | **2.844(1.471-5.497)** |
|  | s-GWG | 0.981(0.937-1.026) | 0.996(0.961-1.033) | 0.997(0.957-1.037) |
|  |  |  |  |  |
| f-NBP | GDM⁺/⁺ | - | **3.731(1.762-7.901)** | **2.635(1.207-5.752)** |
|  | s-GWG | 1.000(0.990-1.010) | 1.000(0.992-1.009) | 1.001(0.993-1.009) |
|  |  |  |  |  |
| f-HDP | GDM⁺/⁺ | - | 3.177(0.950-10.624) | 2.637(0.716-9.710) |
|  | s-GWG | 0.870(0.790-0.958) | **0.873(0.790-0.965)** | **0.877(0.792-0.971)** |
|  |  |  |  |  |
| s-YMA | GDM⁺/⁺ | - | **3.275(1.333-8.048)** | 2.234(0.775-6.443) |
|  | s-GWG | 0.975(0.915-1.040) | 0.985(0.926-1.048) | 0.990(0.926-1.060) |
|  |  |  |  |  |
| s-AMA | GDM⁺/⁺ | - | **3.960(1.862-8.422)** | **3.140(1.349-7.305)** |
|  | s-GWG | 0.990(0.931-1.052) | 0.999(0.982-1.016) | 0.999(0.982-1.018) |
|  |  |  |  |  |
| SIPI | GDM⁺/⁺ | - | 2.666(0.886-8.022) | 1.255(0.322-4.895) |
|  | s-GWG | 0.962(0.887-1.045) | 0.972(0.896-1.054) | 0.967(0.877-1.066) |
|  |  |  |  |  |
| LIPI | GDM⁺/⁺ | - | **4.778(2.442-9.351)** | **3.737(1.750-7.984)** |
|  | s-GWG | 0.986(0.933-1.041) | 0.999(0.980-1.018) | 1.000(0.985-1.015) |
|  |  |  |  |  |
| s-UW | GDM⁺/⁺ | - | 5.608(0.588-53.450) | 4.072(0.341-48.612) |
|  | s-GWG | 1.035(0.947-1.131) | 1.046(0.962-1.137) | 1.043(0.963-1.131) |
|  |  |  |  |  |
| s-NW | GDM⁺/⁺ | - | **2.948(1.205-7.217)** | 1.921(0.676-5.455) |
|  | s-GWG | 0.999(0.983-1.015) | 1.000(0.987-1.012) | 0.997(0.949-1.048) |
|  |  |  |  |  |
| s-OB | GDM⁺/⁺ | - | **3.986(1.737-9.149)** | **4.252(1.595-11.341)** |
|  | s-GWG | 0.971(0.912-1.034) | 0.986(0.929-1.046) | 0.971(0.901-1.046) |

*Abbreviations: f-, first pregnancy; s-, subsequent pregnancy; GDM, gestational diabetes mellitus; GDM^+^/^+^, GDM history with recurrence; GWG, gestational weight gain; HDP, hypertensive disorders of pregnancy; NBP, normal blood pressure; HDP, hypertensive disorders of pregnancy; YMA, young maternal age; AMA, advanced maternal age; SIPI, short interpregnancy interval; LIPI, long interpregnancy interval; UW, underweight; NW, normal weight; OB, overweight/obesity; OR, odds ratio; CI, confidence interval. Model 1: Adjusted for GDM patterns and s-GWG. Model 2: Adjusted for GDM patterns, s-GWG, LIPI, f-HDP, f-PTB, f-CS, s-AMA, s-BMI categories, and s-parity. Numbers with statistical significance were marked in bold.*
