# Supplementary material for: Analysis of the association between history of gestational diabetes mellitus and hypertensive disorders in a subsequent pregnancy: a retrospective cohort study
Source: Front Endocrinol (Lausanne). 2026 Mar 12;17:1736779. doi: 10.3389/fendo.2026.1736779 (PMC13017284; doi:10.3389/fendo.2026.1736779)
Supplement: Supplementary file 3 [file Table3.docx]

**Supplementary Table 3 Comparison of risk factors between the s-PE group and the s-nonPE Group**

| **Risk factors** | **s-PE (n=69)** | **s-nonPE (n=5859)** | **t/Z/Chi-square** | **P** |
| --- | --- | --- | --- | --- |
| continuous variables (x±s, median[Q1-Q3]) |  |  |  |  |
| f-MA(years,) | 29.00(27.00-31.00) | 28.00(26.00-30.00) | -2.716 | **0.007** |
| s-MA(years,) | 35.00(32.00-38.00) | 32.00(30.00-35.00) | -5.185 | **＜0.001** |
| s-BMI(kg/m^2^) | 23.43(20.91-26.4) | 20.96(19.46-22.90) | -5.699 | **＜0.001** |
| f-parity | 1(1-1) | 1(1-1) | -0.894 | 0.371 |
| s-parity | 2(2-2) | 2(2-2) | -0.798 | 0.425 |
| IPI(months) | 58.64(28.94-83.71) | 35.42(20.65-57.24) | -4.325 | **＜0.001** |
| categorical variables |  |  |  |  |
| GDM status[n (%)] |  |  |  |  |
| GDM^-/-^ | 48(69.6) | 4705(80.3) | 4.948 | **0.026** |
| GDM^+/-^ | 3(4.3) | 246(4.2) | 0.004 | 0.951 |
| GDM^-/+^ | 8(11.6) | 609(10.4) | 0.105 | 0.746 |
| GDM^+/+^ | 10(14.5) | 299(5.1) | 12.169 | **＜0.001** |
| f-HDP[n (%)] |  |  |  |  |
| Yes | 25(36.2) | 157(2.7) | 257.981 | **＜0.001** |
| No | 44(63.8) | 5702(97.3) |  |  |
| f-GH[n (%)] |  |  |  |  |
| Yes | 5(7.2) | 59(1.0) | 24.859 | **＜0.001** |
| No | 64(92.8) | 5800(99.0) |  |  |
| f-PE[n (%)] |  |  |  |  |
| Yes | 20(29.0) | 98(1.7) | 260.770 | **＜0.001** |
| No | 49(71.0) | 5761(98.3) |  |  |
| f-PTB [n (%)] |  |  |  |  |
| Yes | 13(18.8) | 303(5.2) | 25.249 | **＜0.001** |
| No | 56(81.2) | 5556(94.8) |  |  |
| f-CS [n (%)] |  |  |  |  |
| Yes | 44(63.8) | 2233(38.1) | 18.975 | **＜0.001** |
| No | 25(36.2) | 3626(61.9) |  |  |
| IPI categories [n (%)] |  |  |  |  |
| SIPI | 21(30.4) | 3009(51.4) | 11.947 | **＜0.001** |
| LIPI | 48(69.6) | 2850(48.6) |  |  |
| s-MA categories [n (%)] |  |  |  |  |
| s-YMA | 31(44.9) | 4285(73.1) | 27.408 | **＜0.001** |
| s-AMA | 38(55.1) | 1574(26.9) |  |  |
| s-BMI [n (%)] |  |  |  |  |
| s-UW | 3(4.8) | 726(13.1) | 3.716 | 0.054 |
| s-NW | 31(50.0) | 3916(70.8) | 12.792 | **＜0.001** |
| s-OB | 28(45.2) | 888(16.1) | 37.914 | **＜0.001** |

*f-, first pregnancy; s-, subsequent pregnancy; GDM, gestational diabetes mellitus; PE, pre-eclampsia in the second pregnancy; nonPE, no pre-eclampsia; MA,maternal age; YMA, young maternal age (less than 35 years old); AMA, advanced maternal age (35 years or more); SIPI, short inter-pregnancy interval (less than 36 months); LIPI, long inter-pregnancy interval (36 months or more); UW, underweight; NW, normal weight; OB, overweight or obesity. GDM^-/-^, no GDM in neither pregnancy; GDM^+/-^, GDM only in the first pregnancy; GDM^-/+^, GDM only in the second pregnancy; GDM^+/+^, GDM both in the first and the second pregnancy. Bold values indicate P <0.05.*
