# Supplementary material for: Analysis of the association between history of gestational diabetes mellitus and hypertensive disorders in a subsequent pregnancy: a retrospective cohort study
Source: Front Endocrinol (Lausanne). 2026 Mar 12;17:1736779. doi: 10.3389/fendo.2026.1736779 (PMC13017284; doi:10.3389/fendo.2026.1736779)
Supplement: Supplementary file 4 [file Table4.docx]

**Supplementary Table 4 Impact of s-GWG on s-PE in unadjusted and adjusted models**

| The analyzed population | Independent variable | unadjusted OR (95% CI) | adjusted OR (95% CI) in Model 1 | adjusted OR (95% CI) in Model 2 |
| --- | --- | --- | --- | --- |
| All | GDM⁺/⁺ | - | 2.345(0.972-5.655) | 1.231(0.471-3.220) |
|  | s-GWG | 0.999(0.986-1.013) | 1.000(0.989-1.011) | 1.001(0.990-1.011) |
|  |  |  |  |  |
| f-NBP | GDM⁺/⁺ | - | 2.398(0.820-7.008) | 1.531(0.505-4.641) |
|  | s-GWG | 1.000(0.990-1.011) | 1.001(0.991-1.010) | 1.001(0.991-1.010) |
|  |  |  |  |  |
| f-HDP | GDM⁺/⁺ | - | 0.999(0.194-5.141) | 0.576(0.077-4.319) |
|  | s-GWG | 0.961(0.860-1.073) | 0.965(0.862-1.081) | 0.983(0.871-1.109) |
|  |  |  |  |  |
| s-YMA | GDM⁺/⁺ | - | 1.047(0.138-7.948) | 0.447(0.050-3.996 |
|  | s-GWG | 0.999(0.963-1.036) | 0.999(0.962-1.037) | 1.002(0.982-1.022) |
|  |  |  |  |  |
| s-AMA | GDM⁺/⁺ | - | 2.305(0.830-6.401) | 1.659(0.546-5.037) |
|  | s-GWG | 1.000(0.987-1.012) | 1.000(0.989-1.011) | 1.000(0.989-1.012) |
|  |  |  |  |  |
| SIPI | GDM⁺/⁺ | - | - | - |
|  | s-GWG | 0.965(0.857-1.087) | 0.965(0.855-1.088) | 0.952(0.823-1.100) |
|  |  |  |  |  |
| LIPI | GDM⁺/⁺ | - | **3.096(1.234-7.767)** | 1.879(0.699-5.051) |
|  | s-GWG | 1.000(0.989-1.011) | 1.000(0.991-1.010) | 1.001(0.992-1.010) |
|  |  |  |  |  |
| s-UW | GDM⁺/⁺ | - | - | - |
|  | s-GWG | 1.052(0.932-1.188) | 1.069(0.949-1.203) | 1.052(0.943-1.172) |
|  |  |  |  |  |
| s-NW | GDM⁺/⁺ | - | 0.843(0.111-6.384) | 0.349(0.040-3.052) |
|  | s-GWG | 1.000(0.991-1.011) | 1.001(0.991-1.010) | 1.000(0.989-1.012) |
|  |  |  |  |  |
| s-OB | GDM⁺/⁺ | - | 2.535(0.873-7.364) | 2.321(0.720-7.481) |
|  | s-GWG | 0.995(0.932-1.063) | 1.003(0.941-1.069) | 0.996(0.925-1.074) |

*Abbreviations: f-, first pregnancy; s-, subsequent pregnancy; GDM, gestational diabetes mellitus; GDM^+^/^+^, GDM history with recurrence; GWG, gestational weight gain; PE, pre-eclampsia; HDP, hypertensive disorders of pregnancy; NBP, normal blood pressure; HDP, hypertensive disorders of pregnancy; YMA, young maternal age; AMA, advanced maternal age; SIPI, short interpregnancy interval; LIPI, long interpregnancy interval; UW, underweight; NW, normal weight; OB, overweight/obesity; OR, odds ratio; CI, confidence interval. Model 1: Adjusted for GDM patterns and s-GWG. Model 2: Adjusted for GDM patterns, s-GWG, IPI, f-HDP, f-PTB, f-CS, s-MA, s-BMI, and s-parity. Numbers with statistical significance were marked in bold.*
