# Supplementary material for: Analysis of the association between history of gestational diabetes mellitus and hypertensive disorders in a subsequent pregnancy: a retrospective cohort study
Source: Front Endocrinol (Lausanne). 2026 Mar 12;17:1736779. doi: 10.3389/fendo.2026.1736779 (PMC13017284; doi:10.3389/fendo.2026.1736779)
Supplement: Supplementary file 5 [file Table5.docx]

**Supplementary Table 5 The influence of GDM classification in stratified subgroups on s-PE**

| Subgroups | OR (95%CI) for the s-PE | | | |
| --- | --- | --- | --- | --- |
|  | GDM⁻/⁻ | GDM⁺/⁻ | GDM⁻/⁺ | GDM⁺/⁺ |
| **unadjusted model** |  |  |  |  |
| f-NBP (n=5746) | reference | 1.137(0.272-4.762) | 0.932(0.329-2.635) | 1.919(0.676-5.447) |
| f-HDP (n=182) | reference | 0.929(0.108-7.991) | 1.061(0.324-3.478) | 2.622(0.886-7.760) |
|  |  |  |  |  |
| s-YMA (n=4316) | reference | 2.725(0.812-9.141) | 0.810(0.191-3.439) | 1.674(0.392-7.137) |
| s-AMA (n=1612) | reference | - | 1.172(0.474-2.899) | **3.113(1.369-7.081)** |
|  |  |  |  |  |
| SIPI (n=3030) | reference | 1.800(0.411-7.893) | 1.460(0.334-6.394) | 1.046(0.138-7.941) |
| LIPI (n=2898) | reference | 0.941(0.127-6.979) | 1.052(0.437-2.533) | **4.150(1.945-8.853)** |
|  |  |  |  |  |
| s-UW (n=729) | reference | 12.231(1.074-139.245) | - | - |
| s-NW (n=3947) | reference | 0.855(0.115-6.369) | 1.383(0.476-4.019) | 2.150(0.64-7.224) |
| s-OB (n=916) | reference | 0.721(0.094-5.539) | 1.025(0.338-3.111) | **3.564(1.42-8.943)** |
| **Model 1** |  |  |  |  |
| f-NBP (n=5746) | reference | 1.332(0.315-5.631) | 0.735(0.259-2.091) | 1.735(0.608-4.950) |
| f-HDP (n=182) | reference | 0.899(0.098-8.220) | 0.922(0.272-3.127) | 2.568(0.841-7.842) |
|  |  |  |  |  |
| s-YMA (n=4316) | reference | 2.746(0.772-9.769) | 0.570(0.130-2.500) | 0.965(0.212-4.398) |
| s-AMA (n=1612) | reference | - | 0.675(0.251-1.813) | 1.989(0.795-4.977) |
|  |  |  |  |  |
| SIPI (n=3030) | reference | 1.238(0.257-5.968) | 0.795(0.168-3.759) | 0.509(0.062-4.172) |
| LIPI (n=2898) | reference | 0.897(0.119-6.767) | 0.818(0.330-2.025) | **2.883(1.285-6.465)** |
|  |  |  |  |  |
| s-UW (n=729) | reference | 19.815(1.349-291.104) | - | - |
| s-NW (n=3947) | reference | 0.982(0.128-7.528) | 0.876(0.289-2.651) | 1.288(0.357-4.650) |
| s-OB (n=916) | reference | 0.647(0.077-5.411) | 0.709(0.214-2.342) | 2.723(0.993-7.468) |
| **Model 2** |  |  |  |  |
| f-NBP (n=5746) | reference | 1.156(0.267-5.011) | 0.645(0.222-1.874) | 1.479(0.505-4.326) |
| f-HDP (n=182) | reference | 0.843(0.079-9.006) | 0.758(0.201-2.859) | 2.120(0.634-7.090) |
|  |  |  |  |  |
| s-YMA (n=4316) | reference | 2.864(0.767-10.692) | 0.519(0.114-2.375) | 0.731(0.148-3.614) |
| s-AMA (n=1612) | reference | - | 0.746(0.275-2.024) | 2.270(0.903-5.711) |
|  |  |  |  |  |
| SIPI (n=3030) | reference | 1.155(0.218-6.124) | 0.690(0.135-3.534) | 0.357(0.036-3.565) |
| LIPI (n=2898) | reference | 0.774(0.101-5.945) | 0.688(0.267-1.771) | **2.486(1.076-5.746)** |
|  |  |  |  |  |
| s-UW (n=729) | reference | 17.416(1.161-261.239) | - | - |
| s-NW (n=3947) | reference | 0.843(0.109-6.509) | 0.702(0.226-2.184) | 0.945(0.252-3.553) |
| s-OB (n=916) | reference | 0.584(0.068-4.997) | 0.647(0.192-2.177) | 2.617(0.947-7.234) |

*GDM, gestational diabetes mellitus; GDM⁻/⁻, no GDM in either pregnancy; GDM^+^/⁻, GDM history without recurrence; GDM⁻/^+^, GDM only in subsequent pregnancy; GDM^+^/^+^, GDM history with recurrence; PE, pre-eclampsia; NBP, normal blood pressure; HDP, hypertensive disorders of pregnancy; OR, odds ratio; CI, confidence interval. Model 1: Adjusted for first-pregnancy factors (GDM patterns, LIPI, f-PE, f-PTB, f-CS). Model 2: Adjusted for factors in Model 1 plus second-pregnancy factors (s-AMA, s-BMI categories, s-parity).*
